# Permethrin-Treated Clothing as Protection against the Dengue Vector, *Aedes aegypti*: Extent and Duration of Protection

**DOI:** 10.1371/journal.pntd.0004109

**Published:** 2015-10-06

**Authors:** Sarah DeRaedt Banks, James Orsborne, Salvador A. Gezan, Harparkash Kaur, Annelies Wilder-Smith, Steve W. Lindsey, James G. Logan

**Affiliations:** 1 Department of Disease Control, London School of Hygiene and Tropical Medicine, London, United Kingdom; 2 *arctec*, London School of Hygiene and Tropical Medicine, London, United Kingdom; 3 SFRC, University of Florida, Gainesville, Florida, United States of America; 4 Department of Global Health and Epidemiology, Umea University, Umea, Sweden; 5 Lee Kong Chian School of Medicine, Nanyang Technological University, Singapore; 6 School of Biological and Biomedical Sciences, Durham University, Durham, United Kingdom; Liverpool School of Tropical Medicine, UNITED KINGDOM

## Abstract

**Introduction:**

Dengue transmission by the mosquito vector, *Aedes aegypti*, occurs indoors and outdoors during the day. Personal protection of individuals, particularly when outside, is challenging. Here we assess the efficacy and durability of different types of insecticide-treated clothing on laboratory-reared *Ae*. *aegypti*.

**Methods:**

Standardised World Health Organisation Pesticide Evaluation Scheme (WHOPES) cone tests and arm-in-cage assays were used to assess knockdown (KD) and mortality of *Ae*. *aegypti* tested against factory-treated fabric, home-dipped fabric and microencapsulated fabric. Based on the testing of these three different treatment types, the most protective was selected for further analysis using arm-in cage assays with the effect of washing, ultra-violet light, and ironing investigated using high pressure liquid chromatography.

**Results:**

Efficacy varied between the microencapsulated and factory dipped fabrics in cone testing. Factory-dipped clothing showed the greatest effect on KD (3 min 38.1%; 1 hour 96.5%) and mortality (97.1%) with no significant difference between this and the factory dipped school uniforms. Factory-dipped clothing was therefore selected for further testing. Factory dipped clothing provided 59% (95% CI = 49.2%– 66.9%) reduction in landing and a 100% reduction in biting in arm-in-cage tests. Washing duration and technique had a significant effect, with insecticidal longevity shown to be greater with machine washing (LW_50_ = 33.4) compared to simulated hand washing (LW_50_ = 17.6). Ironing significantly reduced permethrin content after 1 week of simulated use, with a 96.7% decrease after 3 months although UV exposure did not reduce permethrin content within clothing significantly after 3 months simulated use.

**Conclusion:**

Permethrin-treated clothing may be a promising intervention in reducing dengue transmission. However, our findings also suggest that clothing may provide only short-term protection due to the effect of washing and ironing, highlighting the need for improved fabric treatment techniques.

## Author Summary

Personal protection technologies could be a key tool in the fight against arthropod borne diseases. Insecticide treated clothing may have a significant effect on reducing mosquito borne disease by reducing biting rates and local vector populations. Currently there are four main treatment techniques; absorption, incorporation, polymer coating, and micro-encapsulation which are used to treat clothing with permethrin but little is known regarding the efficacy and duration of protection provided by these techniques. The evaluation of these different treatment techniques using standardised World Health Organisation Pesticide Evaluation Scheme (WHOPES) assays and high pressure liquid chromatography (HPLC) analysis provides further information on factors which have a significant effect on the efficacy and duration of protection of treated clothing. This will aid in the design and implementation of control programs using insecticide treated clothing. However, washing technique and heat exposure have a significant effect on efficacy, emphasising the need for further investigation into treatment techniques, so duration of protection can be increased.

## Introduction

Dengue is the “most important mosquito-borne viral disease” [1] with over 3.5 billion people at risk of infection [2], 50–100 million new infections and 20,000 deaths annually [1]. Most control strategies target the main urban vector, *Aedes aegypti* [3], but despite multiple control programmes designed to reduce the immature and adult stages of the mosquito [4], *Ae*. *aegypti* has continued to increase its distribution over the past 25 years [5]. Since *Ae*. *aegypt*i is a day-biting mosquito, technologies that are worn during the day may offer protection against mosquito bites and have the potential to reduce dengue transmission [6].

Personal protection technologies function by repelling or killing the vector, or by providing a physical barrier between vector and host [7,8]. The best known personal (and community) protection technology is long lasting insecticidal nets (LLIN) commonly used in malaria control programmes [9]. LLIN’s are most effective against mosquitoes that feed during the night, but surprisingly have been shown to provide some protection against day biting vectors such as *Ae*. *aegypti* presumably because they may rest on treated netting [10]. Insecticide-treated clothing is an intervention that could protect individuals during the day, when users are at work or school, and could easily integrate into everyday routines. Indeed, agricultural and wildlife groups, commercial companies and the military currently use insecticide treated clothing [11–13]. A recent modelling study demonstrated that insecticide-treated school uniforms can reduce dengue burden by up to 50% in school children in Thailand, highlighting the potential impact insecticide treated clothing could have on dengue transmission [14].

For the clothing to be a sustainable intervention it must be safe, effective and long-lasting. It must also be able to withstand regular washing, be low—cost and acceptable to members of the local communities. All these factors are influenced by the active ingredient and the type of treatment method. Permethrin, a commonly used pyrethroid, is the main compound used in treated clothing [15]. There are several techniques used currently for treating material with an insecticide. They include absorption, incorporation, polymer coating, and micro-encapsulation [16]. Despite the common use of permethrin-treated clothing in the military and recreational industries, studies have been published on its ability to knockdown (KD), kill, repel and prevent biting [15] but they have not investigated variation between clothing treatment techniques. Other important undetermined factors that might affect efficacy of the treated clothing include repeated washing, exposure to Ultra-Violet (UV) light and heat exposure (for example, caused by ironing). These factors could have a significant effect on the efficacy and duration of protection provided by impregnated clothing when used in the field.

The aim of this study was to investigate the efficacy and duration of protection provided by permethrin-treated clothing which could be later used in a randomised controlled trial of permethrin-treated school uniforms to protect children from Dengue fever in Thailand [17].

## Materials and Methods

### Clothing Preparation

The following types of clothing were used in this study:

1. Factory-dipped clothing (FDC): Long sleeved, 100% cotton beige treated shirts (permethrin polymer coated factory dipped, 0.52% w/w permethrin, Insect Shield)
2. Home dipped clothing (HDC): Long sleeved, 100% cotton untreated beige shirts, provided by Insect Shield dipped with Sawyer (Sawyer Products Inc) permethrin home dipping kits (0.50% permethrin as active ingredient).
3. Microencapsulated clothing (MC): Long sleeved, synthetic blend, light grey shirts (permethrin microencapsulated method, 0.50% w/w Craghoppers).
4. Factory dipped school uniforms (FDSU): Standard cotton, short sleeve shirt/shorts/skirt were selected for testing from traditional Thai school uniforms from Chachoengsao province. These were sent to Insect Shield to be treated (permethrin 0.52% w/w permethrin, Insect Shield). The uniforms were then sent to The London School of Hygiene and Tropical Medicine (LSHTM, UK) to be tested.

### Mosquitoes

*Aedes aegypti*, (pyrethroid susceptible strain) were obtained from reference strain (originally from West Africa, colonised in 1926 with field additions in 1976) held at LSHTM, UK. All mosquitoes were reared and housed under optimal environmental conditions of 25°C ± 2°C and 80% RH with a 12: 12 hour photoperiod. Ages ranged for all testing from 3–7 days old (3–5 for cone tests and 5–7 for arm-in-cage). All mosquitoes used in experiments were nulliparous females fed on 10% glucose solution.

### Testing Summary

#### Clothing evaluation overview

Firstly, comparative WHOPES [18] cone tests were performed to determine the effect of the clothing on knockdown (KD) and mortality of *Ae*. *aegypti* mosquitoes. Three different types of new, unwashed, treated clothing were tested and compared with corresponding untreated controls: Factory-dipped clothing (FDC), Factory dipped school uniforms (FDSU) and Microencapsulated clothing (MC). This experiment was done to determine a) whether there was a significant difference between clothing sourced directly from the manufacturer and Thai School uniforms (with no significant difference, the readily available manufacturers clothing could be used for further experimentation, rather than the more scarce Thai uniform); and b) whether there was a significant difference between factory-dipped clothing and microencapsulated clothing.

Arm-in-cage repellency testing, modified from the WHOPES protocol [19], was also performed to determine the protective efficacy of the clothing. This included FDC, MC and in addition Home dipped clothing (HDC). (FDSU were omitted from the arm-in-cage tests based on the results of initial cone tests (see below). HDC was only evaluated using the arm-in-cage assay due to difficulties in the availability of home dipping kits. Additionally, as the hand dipping technique could only provide protection of up to 5 washes (according to the manufacturer label claims), it would be unlikely to be recommended as a long term treatment method of insecticide treated clothing.

**Fig 1:**
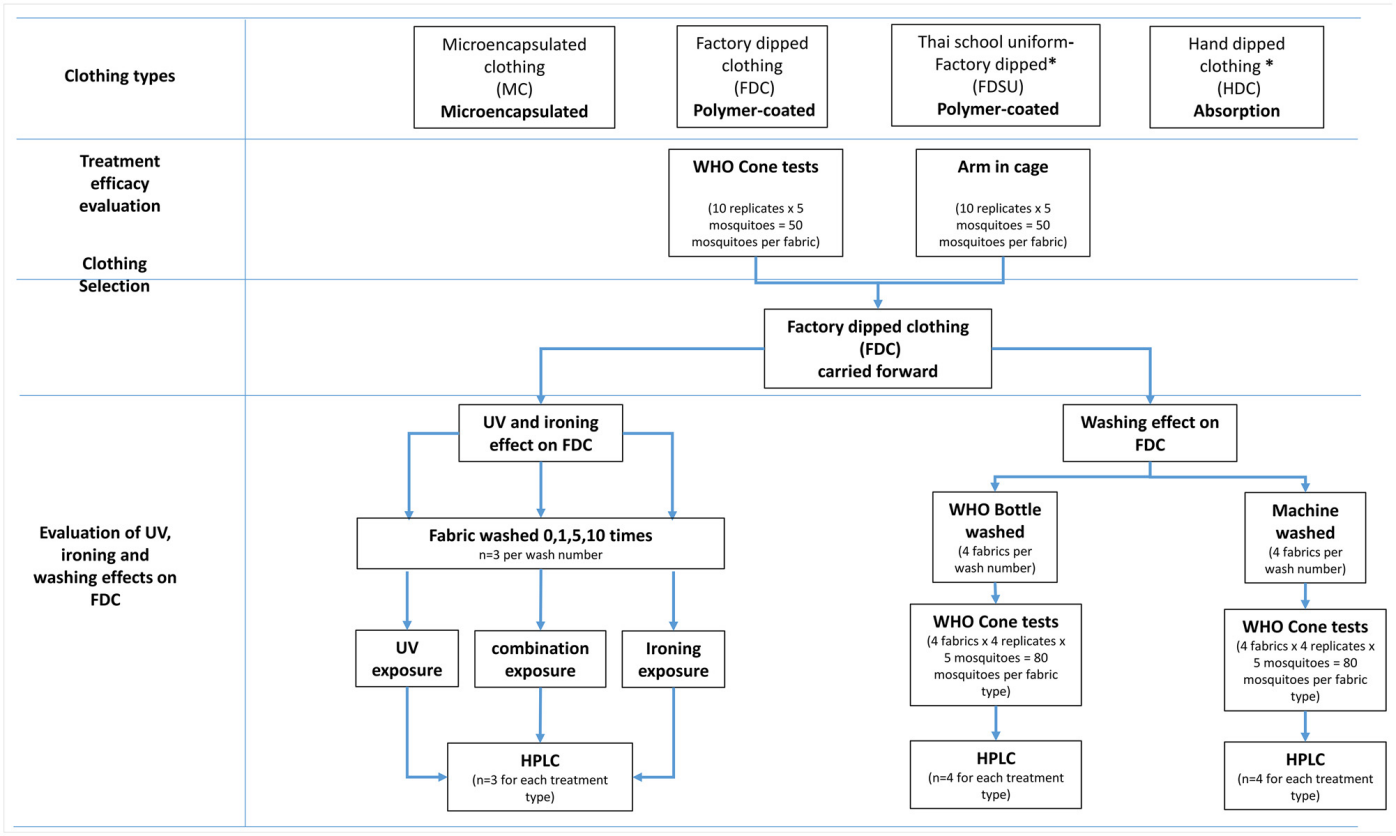
Flow chart of study design and experiments performed. *Denotes that the treatment technique was not included in all efficacy evaluation tests. FDSU was not included in Arm-in-cage assays and HDC was not included in the cone assays.

Following the initial cone and arm-in-cage tests to compare different treatment types, residual activity was evaluated on the FDC only. FDC materials were washed 1, 5, 10, 15, 20, 25, 30 times using a) the WHOPES bottle washing method [18] and b) machine washing. This comparison was important because some residents in Thailand hand-wash their clothes, whilst others use washing machines. Cone assays were then performed on these materials with KD and mortality recorded and HPLC analysis performed to quantify permethrin content within the washed fabrics. FDC clothing was also washed and exposed to ironing, ultraviolet light (UV) or both UV and ironing in combination, for varying degrees of time to simulate field use, then analysed by HPLC to quantify permethrin content. A summary of the testing is provided in Fig 1.

*Cone tests*. Knockdown (KD) and mortality were measured by standardised WHOPES bioassay cone tests [18]. Pieces of material used were 30cm^2^ instead of the WHO standard 25cm^2^ as the material was used also for arm-in-cage testing and needed to be large enough to cover a forearm in subsequent experiments. Pieces of material were secured to a ceramic tile using masking tape. A WHO plastic cone was then secured to the upper side of the tile using rubber bands. Batches of five female mosquitoes were placed in the cone using a mouth aspirator fitted with a high-efficiency particulate arrestance (HEPA) filter and a small cotton plug was used to close the aperture. Bioassays were carried out at 25 ± 2°C and 75 ± 10% relative humidity (RH) following WHOPES guidelines for performing cone tests under laboratory conditions [18]. Mosquitoes were exposed to the materials for three minutes and removed using a mouth aspirator fitted with a HEPA filter. The mosquitoes were then placed in a holding cup inside a humidity chamber (25 ± 2°C and 75 ± 10%) with a net secured over the top with two elastic bands and cotton wool soaked in 10% glucose. KD was recorded 3 minutes and one hour post exposure, with mortality recorded after 24 hours. For each treatment, a corresponding control was performed using untreated fabric. An additional negative control using an untreated tile and a positive control of 0.75% permethrin-treated paper, supplied by a WHO approved lab (Universiti Sains Malaysia, Penang, Malaysia), was included in each set of tests. If a negative control yielded greater than 20% mortality, the experimental replicate was excluded from the analysis and repeated. For initial testing ten replicates were carried out for each sample (10 replicates x 5 mosquitoes = 50 mosquitoes per fabric). For investigating washing effects sixteen replicates were carried out on four pieces of fabric (4 x 4 replicates x 5 mosquitoes = 80 mosquitoes per fabric type).

*Arm-in-cage repellency tests*. Repellency and bite protection was measured by wrapping the forearm of a single participant in unwashed control or treated clothing: FDC, MC and HDC. The material was wrapped around the arm and taped in place. The test consisted of a forearm covered fully by the treated clothing (FCT) with controls of 1) full coverage untreated (FCC), 2) bare arm (BA), and 3) 20% DEET (1.67mg/cm^2^) applied to the forearm.

Thirty female mosquitoes were used for each test. Before each replicate, biting pressure was checked by placing one bare arm with a glove on the hand into the cage for up to 30 seconds. If fewer than ten mosquitoes landed in that time, the cage was refreshed with new mosquitoes. After each test, blood fed mosquitoes were identified and replaced before the following bite pressure test was performed. Once a satisfactory biting pressure was achieved, the test material was placed on the forearm and inserted into the cage with a glove on the hand. The arm remained in the cage for 90 seconds. At the end of 90 seconds, the number of mosquitoes probing on the arm was counted, and two minutes after the test, the number of visible bites (wheals) on the arm were counted to confirm the bites. The forearm was washed with fragrance free soap (Simple Soap, Boots, UK) and water, and wiped down with 75% ethanol after each treatment was tested. The volunteer had one hour of “recovery” time between testing. Bite reactions still present on the volunteers arm after the recovery period were marked with a coloured indelible pen to avoid being counted more than once. A total of ten replicates of 30 mosquitoes each (n = 300) were conducted per treatment based on a sample size requirement to detect a 50% repellent effect. All treatments were tested on one volunteer, in the same day using a Latin square design.

For the arm–in-cage experiments, efficacy was defined by protection against landing and probing (biting). Protection was determined by recording the number of mosquitoes landing or probing on the arm at the end of the 90 second exposure for each treatment (FCT) as a percentage of the number of mosquitoes landing on the control arms (FCC or BA). For example, to determine the protection of an arm fully covered with treated material (FCT) in comparison to an arm fully covered with untreated control material (FCC), the formula below was used. The formula was adapted for each treatment/control comparison as appropriate.

$$Protection(\%)=\left[\frac{FCC-FCT}{FCC}\right]*100$$

Where FCC = Full coverage control and FCT = Full coverage treated

#### Clothing wash methods

The WHO [18] standardized washing tests were performed to simulate hand washing and to provide a repeatable and comparable method for future studies. We compared two washing methods: WHO washing (to simulate hand washing) [18] and washing with a laundry machine. Washing was performed on FDC materials only.

*WHOPES bottle washing*. Pieces of FDC material (30cm^2^) were cut from 4 different treated and untreated shirts for each wash group. Wash groups consisted of eight pieces of material (four treated and four untreated) washed 0, 1, 5, 10, 15, 20, 25 and 30 times. The WHO washing protocol [18] is as follows: Savon de Marseille soap was dissolved in distilled water at 2g/litre. 500ml of the water/soap mixture is poured into 1 litre bottles. The clothing to be washed was placed and submerged into the 1 litre bottles with the water/soap mixture. Bottles were then placed into a 30°C shaking water bath (set to 155 RPM) for 10 minutes. After 10 minutes, bottle were removed from the water bath and water/soap mixture removed, the bottle was then filled with 500ml of clean distilled water. Bottles were placed back into a 30°C shaking water bath (set to 155 RPM) for another 10 minutes. Clothing was then removed and left to dry at 30°C for 45 minutes.

*Machine washing*. Wash groups consisted of eight pieces of FDC permethrin-treated material (four treated, four untreated). These were washed 0, 1, 5, 10, 15, 20, 25, 30 times. A Hotpoint washing machine (model WMSL 521P) was used to wash the material. Separate machines were used for washing treated and untreated materials. The machines were set for a 30 minute cycle with a water temperature of 30°C and a spin speed of 800 rpm. Violet’s (Homescents, UK) natural unscented washing powder was used as the detergent with one scoopful (25 ml) used for every wash, in accordance with detergent to water volume. Each full wash used 59 L of water. This wash process was repeated for each wash group until the appropriate number of washes was reached.

#### Ultra-violet (UV) and ironing exposure

Square pieces (5 cm^2^) of factory dipped clothing (FDC) were either exposed to UV light or ironed, or both in combination. Irradiation with UV was performed by exposing the clothing from above with an OSRAM UV-sun radiation lamp (300 W, Ultra Vitalux), which emits UV radiation simulating sunlight. The lamp is designed to provide UV radiation equivalent to mid-day natural sunlight (approximately 1KW/m^2^) at a distance of 50 cm from a surface. The lamp was adjusted to 12.5 cm to provide a radiation intensity 16 times that of normal mid-day sunlight and exposure time set to 20, 100, 400 and 1200 minutes. Ironed clothing was exposed to an iron (surface temperature 200°C) for 30 seconds 0, 1, 2, 8 and 24 times. Both of these regimes were chosen to simulate 1 day, 1 week, 1 month and 3 months use respectively, assuming the clothing was ironed twice per week and worn five days per week. This process was repeated for pieces of clothing washed 0, 5, 10 and 20 times. The combination of UV and ironing exposures was the same as above except clothing was washed 1, 2, 8 and 24 times to reflect a more realistic use of the clothing, with the assumption of clothing being washed and ironed twice per week and the clothing worn for 5 days per week. Three replicates were performed for each treatment type and permethrin content was analysed by HPLC.

#### High performance liquid chromatography (HPLC)

HPLC analyses were carried out using a Dionex Ultimate 3000 range of equipment and software (Camberley, Surrey, UK). Samples were separated on an Acclaim C_18_ 120 Ǻ (250 x 4.6 mm, Dionex, UK) column eluting with water/acetonitrile (90:10%; v/v) at a flow rate of 2 ml/min and passed through the photodiode array detector (PDA-100, Dionex) set at 275 nm. The authenticity of the detected peaks was determined by comparison of retention time, spectral extraction at 275 nm and spiking the sample with commercially available standard of the insecticide. A calibration curve of insecticide was generated by Chromeleon (Dionex software) using known amounts of the standard (0–0.4 ug/ml) in acetonitrile injected onto the column. From this curve the amount of insecticide in the matrix was calculated. Doses of insecticide per m^2^ were calculated from the quantities detected in each of 2.5cm^2^ pieces. Two pieces (2.5cm^2^) were evaluated from each replicate for all experiments resulting in 6 pieces from 3 replicates for the UV and ironing exposure and 8 pieces from 4 replicates for each washing types for the washed fabrics.

### Statistical Analysis

#### Arm–in-cage and cone assay analysis

A generalised linear model was fitted for each experiment based on a binomial distribution with a logic link (i.e. logistic regression). Factors included temperature, humidity and time of day. Only factors which were shown to have a significant effect were used in the final model. Comparisons between predicted means for each treatment type were performed using an LSD post-hoc test with a significance level set at 5%. The binary responses for cone assays included 3 min, 1hr knockdown (KD) and 24hr mortality as well as biting and landing where appropriate. For investigating the effect of different washing techniques, the wash number which represented 90% and 50% KD and mortality were calculated and referred to in the text as lethal wash 50 and lethal wash 90 (LW_50_ and LW_90_) for both washing techniques. All models were fitted using PROC GLIMMIX implemented in SAS/STAT software, Version 9.3.

#### HPLC analysis

A generalized linear model was fitted for each experiment. Predicted means were compared using an LSD post-hoc test with a significance level set at 5%. Models were fitted using IBM SPSS Version 20 (IBM Corp. Released 2011).

### Ethics Statement

This study was approved by the London School of Hygiene and Tropical Medicine Ethics committee (reference number 6074). The participant used in this study was aged between the ages of 18–65 and provided written informed consent before taking part in this study.

## Results

### Treatment Technique Evaluation

No difference in knockdown after 3 minutes was shown between factory-dipped clothing (FDC), factory dipped school uniforms (FDSU) and microencapsulated clothing (MC) (Table 1). After 1 hour exposure, the FDC and FDSU produced a knockdown of 96.5% (95% CI 94.2%-97.8%) and 98.2% (95% CI 92.1%-99.6%) respectively (Table 1). MC produced a lower 1 hour knockdown, 50.8% (95% CI 37.5%-62.7%) and 24 hour mortality, 73.3% (95% CI 51.8%-87.4%) (Table 1) when compared to both FDC (1 hour KD, p = 0.0043, 24 hour mortality p = 0.0331) and FDSU (1 hour KD, p = 0.0089 and 24 hour mortality, p = 0.0384). There was no difference between FDC and FDSU across any of the time points.

**Table 1:** Standardised WHOPES cone test using three clothing types Factory-dipped clothing (FDC), Micro-encapsulated (MC), Factory-dipped school uniform (FDSU). Knockdown (KD) at 3 minutes, 1 hour post exposure and mortality 24 hours post exposure were recorded and are displayed with 95% confidence intervals.

Clothing type	Percentage KD, 3min (95% CI)	Percentage KD, 1 hour (95% CI)	Percentage Mortality, 24 hours (95% CI)
**Factory-dipped clothing (FDC)**	38.1 (23.2–55.5)	96.5 (94.2–97.8)	97.1 (80.9–99.6)
**Micro-encapsulated (MC)**	20.9 (10.5–37.2)	**50.8 (37.5–62.7)** *	**73.3 (51.8–87.4)** *
**Factory-dipped school uniform(FDSU)**	35.9 (24.9–48.4)	98.2 (92.1–99.6)	98.6 (80.6–99.9)

* = difference between clothing types is significant at the 0.05 level

### Repellency Evaluation

**Fig 2:**
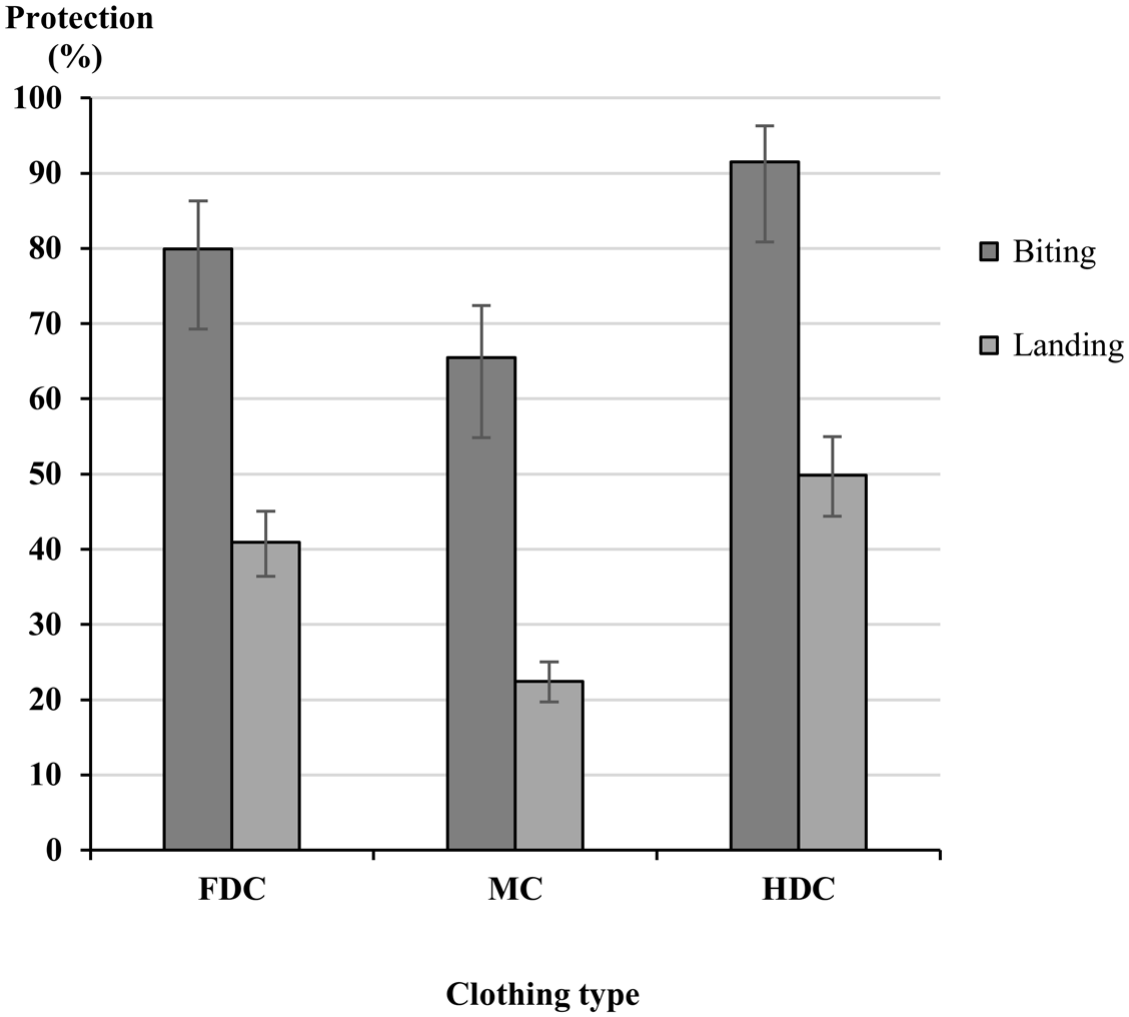
WHOPES arm-in-cage biting and landing full coverage protection for three different clothing impregnation methods; factory-dipped clothing (FDC), micro-encapsulated (MC), hand dipped clothing (HDC). Protection is measured against the control clothing (FCC) for both biting and landing, Whiskers correspond to 95% confidence intervals.

There was a significant difference between mean number of mosquitoes landing between bare arm, control clothing and treated clothing (p = 0.0001). Control and treated clothing did provide significant protection against landing and biting when compared to the bare arm control (p = 0.0001), with the exception of the microencapsulated (MC) untreated control clothing where there was no significant difference between the control clothing and bare arm (p = 0.5776) (Fig 2). Bite protection for MC was significantly lower at 65.5% (95% CI 57.7%-72.4%) compared to HDC 91.5% (95% CI 80.9%- 96.3%), (p<0.0001), (Fig 2). MC gave a bite protection of 79.9% (95% CI 70.41%-86.32%) and this is not significantly different when compared to FDC (p = 0.538). No significant difference was found between HDC and FDC for both landing and biting protection (HDC bite protection: 91.5, 95% CI 80.9%-96.3%); HDC landing protection: 49.9% (95% CI 44.4%- 55.0%); FDC bite protection: 79.9% (95% CI 70.41%- 86.32%); FDC landing protection 40.9% (95% CI 36.4%-45.1%) (p = 0.999), with both FDC and HDC providing the greatest protection against both landing and biting.

### Washing Evaluation of Factory Dipped Clothing (FDC) Using WHO Cone Tests

#### WHO washing technique

**Fig 3:**
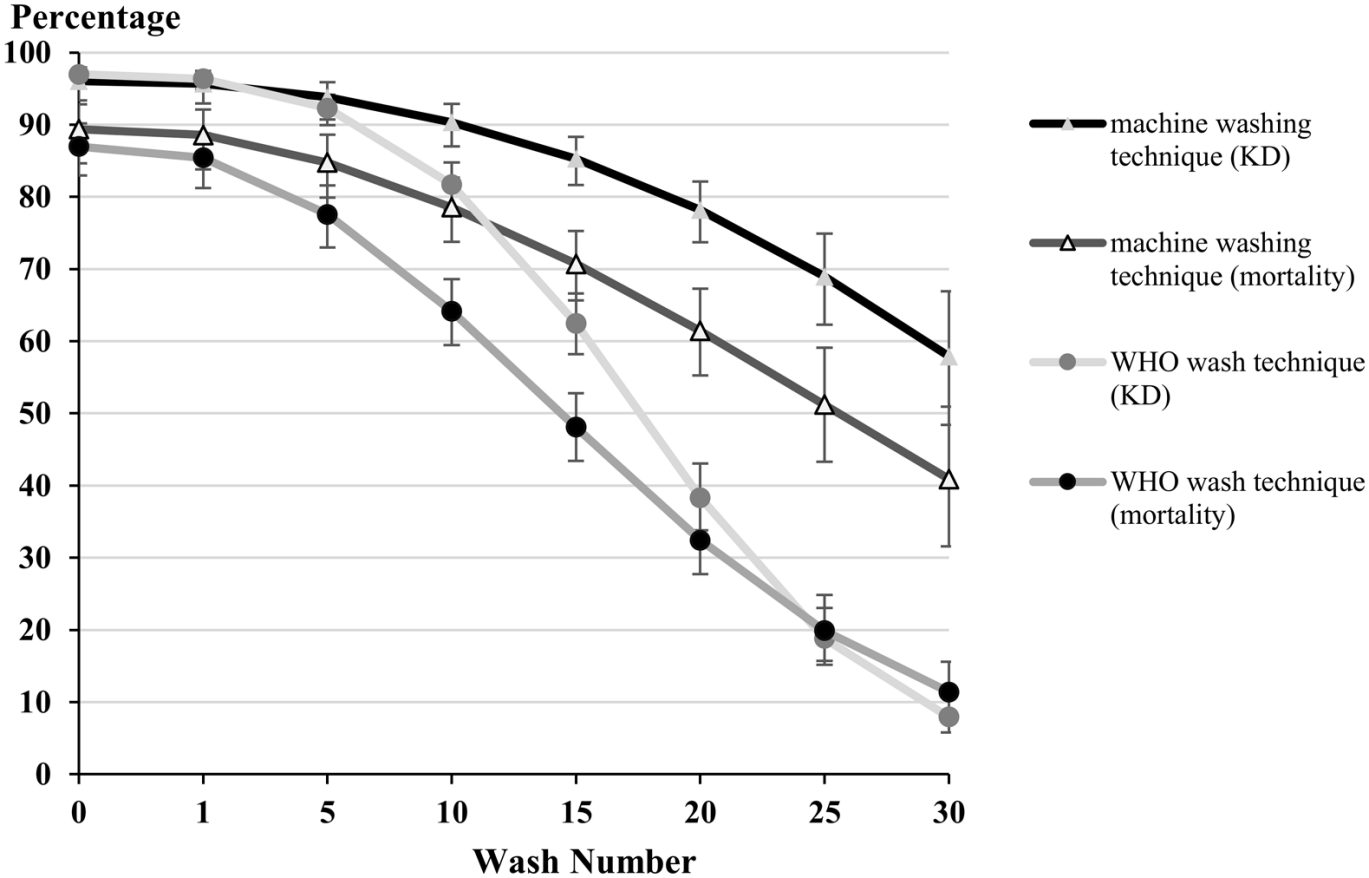
One hour knockdown and 24 hour mortality of factory dipped clothing after using WHO and machine washing techniques after a 3 minute exposure to clothing. Whiskers correspond to 95% confidence intervals

Knockdown (KD) and mortality for unwashed insecticide-treated material were 96.9% and 86.9% respectively with 80% mortality being maintained for up to one wash and > 80% KD maintained for up to 10 washes (Fig 3). From 10 to 20 washes, KD decreased from 81.7% to 38.3% respectively. Mortality also decreased as wash number increased, with 77.5% mortality at 5 washes dropping to 32.4% after 20 washes. After 30 washes 7.9% KD and 11.4% mortality was achieved (Fig 3).

#### Machine washing technique

Knockdown and mortality for unwashed insecticide-treated material were 96.0% and 89.3% respectively. KD was maintained above 80% for up to 15 washes and mortality was maintained above 80% for up to 5 washes (Fig 3). Knockdown and mortality decreased as the number of washes increased, with 90.3% KD and 78.5% mortality at 10 washes and 78.21% and 61.42% at 20 washes. After 30 washes KD and mortality were 57.9% and 40.9% respectively (Fig 3).

No significant differences were observed when washed clothing was compared after one wash. However, mortality and KD were significantly lower after the fifth wash (p = 0.0257) and KD after the 10^th^ wash (p = 0.0003). The differences between knockdown and mortality of the two washing techniques at washes 15–30 were highly significant at all wash points (p<0.0001). 90% knockdown was maintained up until the 10^th^ wash for the machine washed clothing, KDW_90_ 10.0 (95% CI 6.1–13.4) and up until the 6^th^ wash for the WHO method washed clothing (KDW_90_ 6.4; 95% CI 5.0–7.7) (Fig 3). 90% mortality was not achieved for any of the treatments and therefore the LW_90_ was not calculated. The KDW_50_ was 33.4 washes (95% CI 29.5–39.5) for the machine washed clothing, and 17.6 (16.7–18.5) for WHO technique washed clothing. Finally, 50% mortality was obtained with LW_50_ of 14.4 (95% CI 13.0–15.9) washes with the WHO technique and 25.6 (95% CI 22.2–30.6) washes with the machine washing technique (Fig 3).

### High Pressure Liquid Chromatography (HPLC) Analysis

#### Washing effect

**Fig 4:**
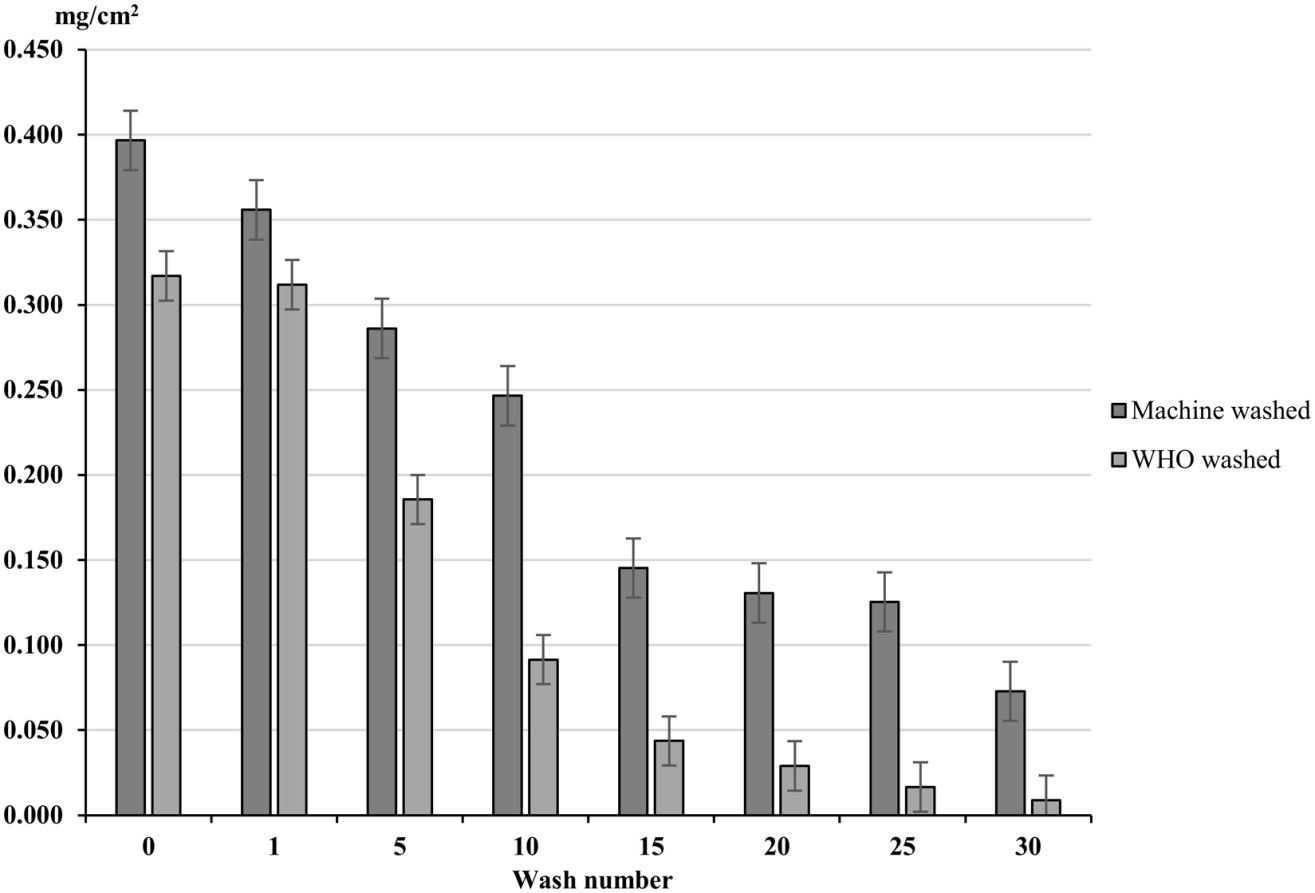
Permethrin content (mg/cm^2^) of WHO bottle and machine washed materials at 0, 1, 5, 10, 15, 20, 25 & 30 washes. Whiskers correspond to 95% confidence intervals.

The HPLC analysis performed on factory dipped (FDC) clothing demonstrated that the concentration of permethrin on treated clothing decreased with washing. For the WHO washing technique, permethrin concentration decreased from 0.317 mg/cm^2^ for unwashed clothing to 0.186 mg/cm^2^ after five washes (p<0.0001 a 41.3% decrease) (Fig 4), and to a concentration of 0.009 mg/cm^2^ after 30 washes (p<0.0001 a decrease of 97.2%). The machine washing technique produced a decrease from 0.397 mg/cm^2^ to 0.286 mg/cm^2^ after five washes (p<0.0001, a decrease of 28%) with 0.073 mg/cm^2^ of permethrin present after 30 washes (a decrease of 81.6%) (Fig 4).

#### Ironing and ultra-violet effect

**Fig 5:**
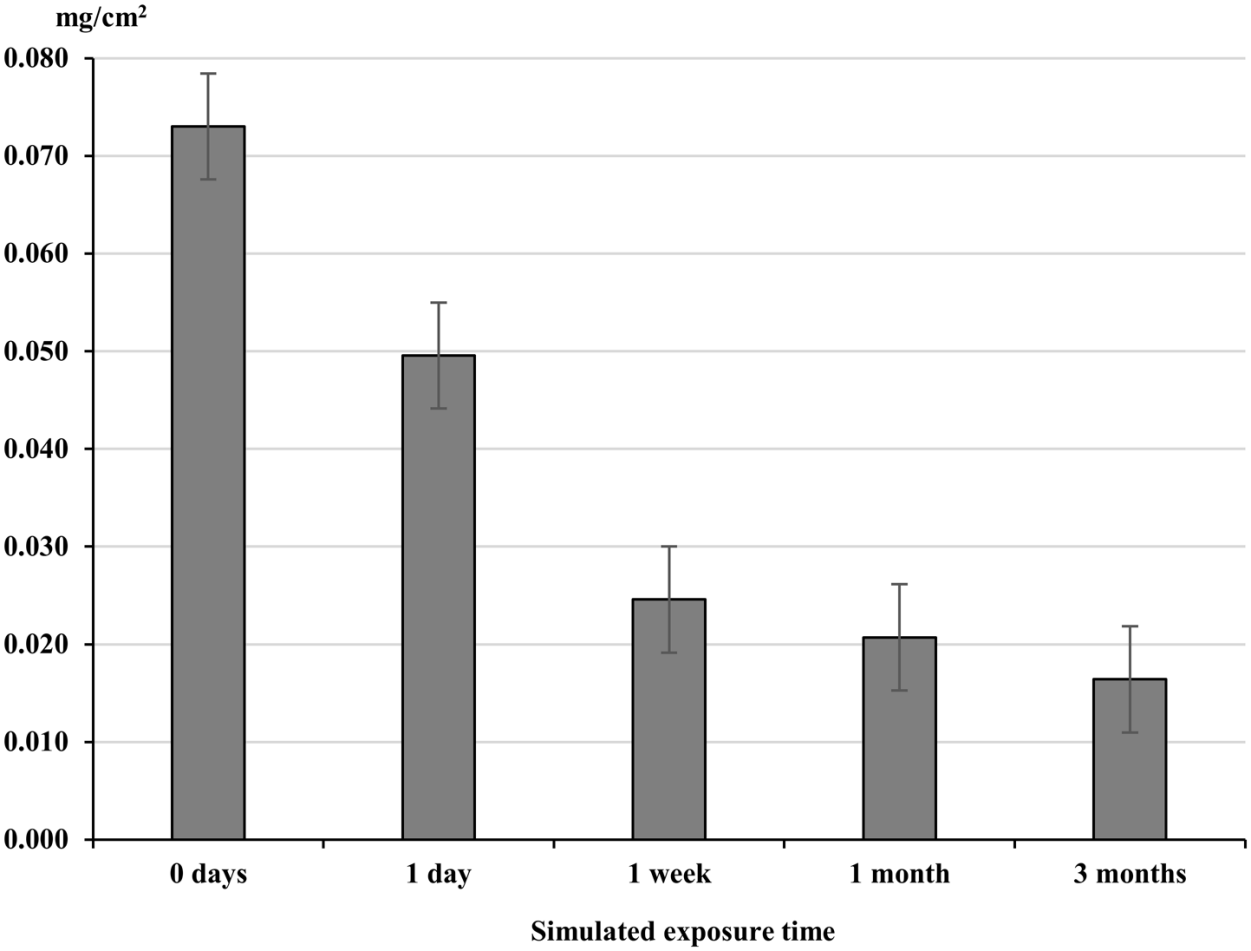
Permethrin content (mg/cm^2^) of treated material exposed to UV-light, ironing and washing in combination. Whiskers correspond to 95% confidence intervals. Clothing was exposed to a combination of UV, Ironing and Washing in combination; 1 day (1 wash, 20min UV, 30 seconds Ironing); 1 week (2 washes, 100 min UV, 1 minute Ironing), 1 month (8 washes, 400 min UV,4 minutes ironing), 3 months (24 washes, 1200 min UV, 12 minutes ironing).

The combination of UV light and ironing on washed clothing reduced the permethrin concentration within factory dipped clothing (FDC) with significant decreases at one day, one week and one month simulated exposure (Fig 5). One day’s simulated use caused a significant decrease from 0.073 to 0.05 mg/cm^2^ (p = <0.0001), a decrease of 31.5%. After three months simulated use, permethrin concentrations were 0.016 mg/cm^2^—a decrease of 78.1%.

**Fig 6:**
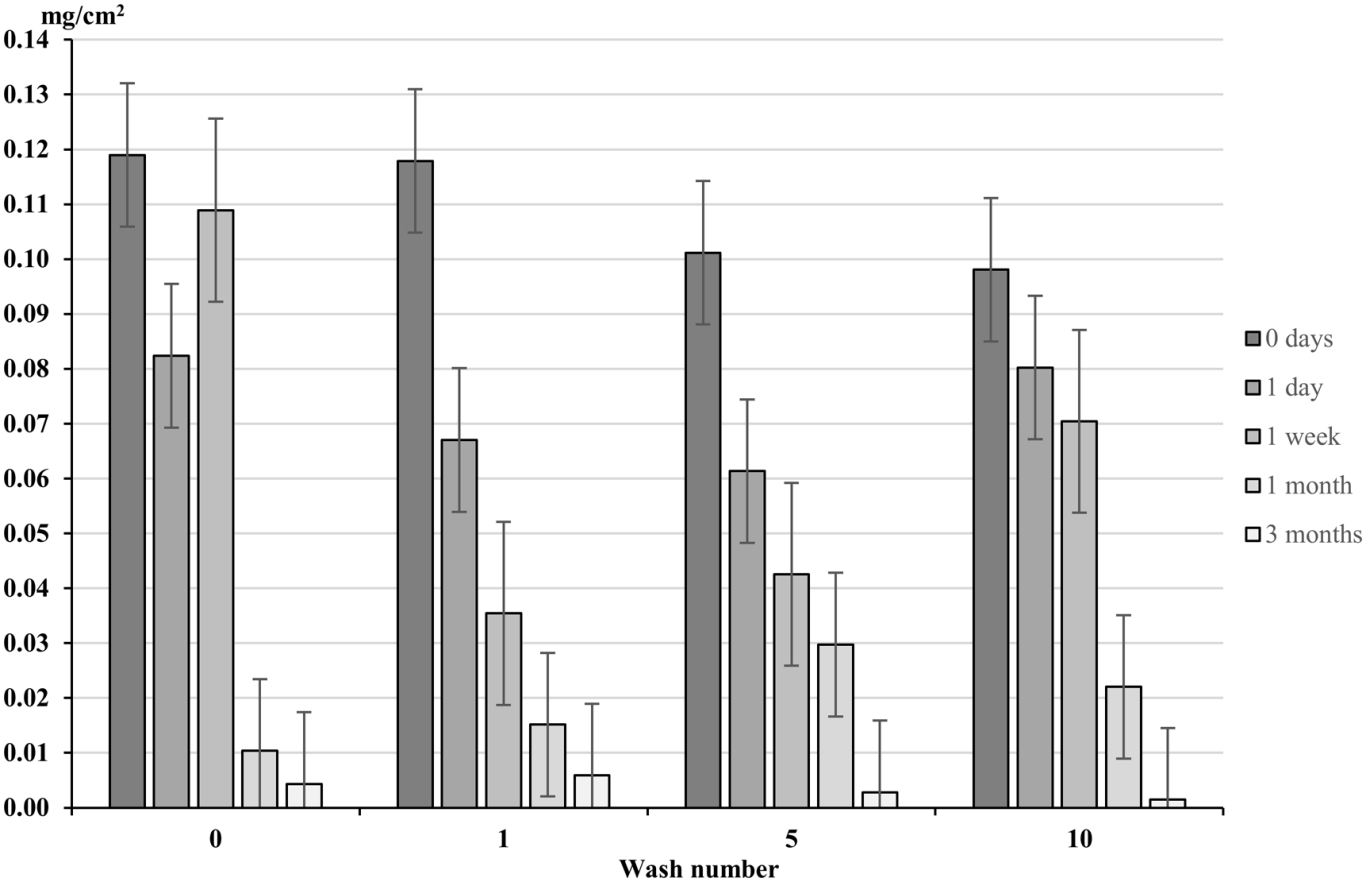
Permethrin content (mg/cm^2^) of treated material exposed to ironing. Whiskers correspond to 95% confidence intervals. Clothing was exposed to Ironing for 0 seconds, 30 seconds, 1 minute, 4 minutes and 12 minutes to simulate 0, 1 day, 1 week, 1 month and 3 months exposure.

Ironing alone caused a decrease in permethrin concentration at all wash points (Fig 6). For unwashed material, permethrin concentration decreased significantly across all wash points after one and three months simulated exposure. Permethrin concentration decreased from 0.119 mg/cm^2^ at day 0 to 0.004 mg/cm^2^ (a 96.7% decrease) after 3 months simulated exposure (p>0.0001) (Fig 6). After 3 months of simulated ironing and ten washes, permethrin concentration was measured at 0.001 mg/cm^2^ (Fig 6).

**Fig 7:**
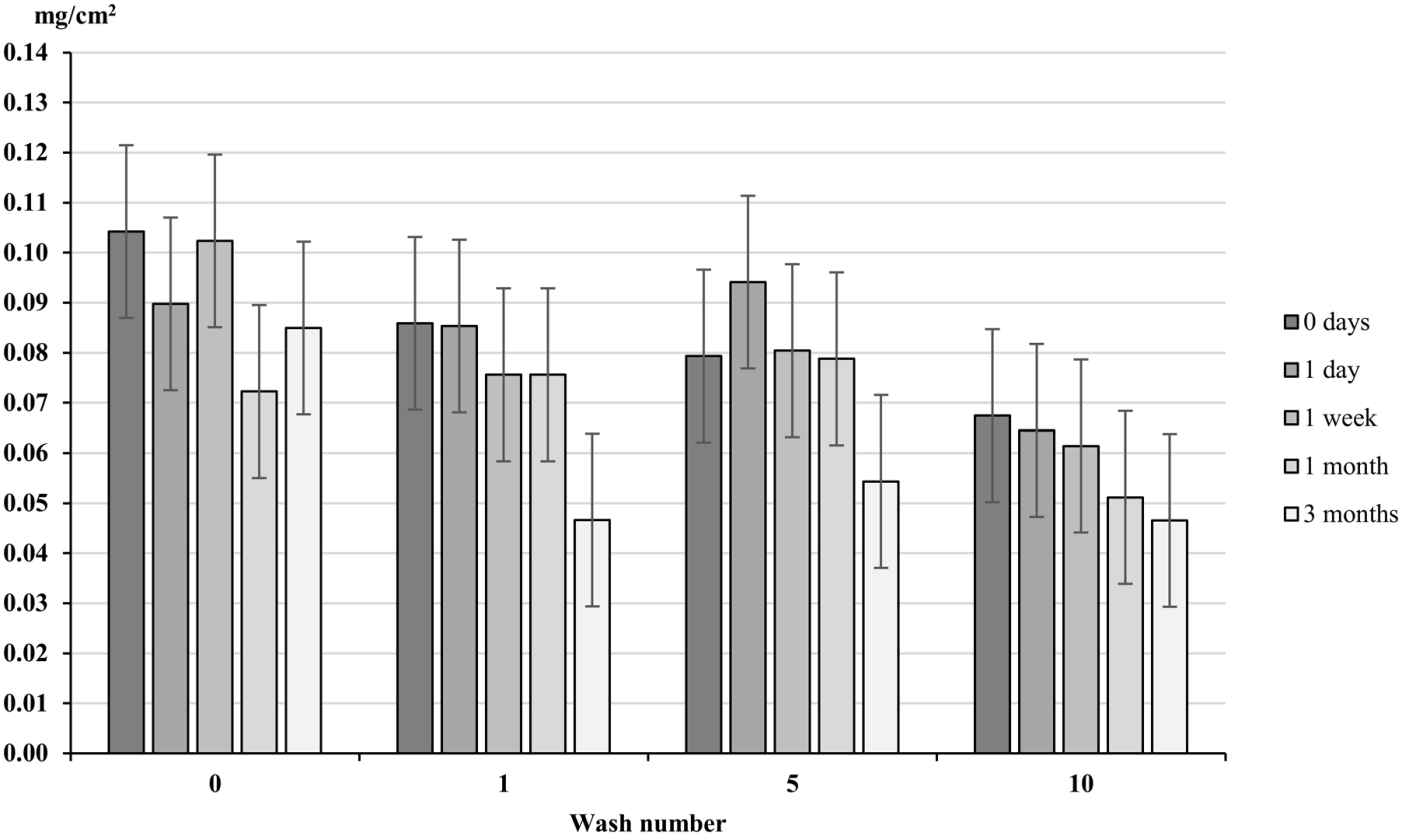
Permethrin content (mg/cm^2^) of treated material exposed to UV-light. Whiskers correspond to 95% confidence intervals. Clothing was exposed to UV-light for 0 seconds, 30 seconds, 1 minute, 4 minutes and 12 minutes to simulate 0, 1 day, 1 week, 1 month and 3 months exposure.

Increased exposure to UV light gave no significant decrease in concentration of permethrin across all washes with the exception of unwashed material and wash 1 (Fig 7). Unwashed material had a significant decrease of 0.032 mg/cm^2^ (30.6%) after 1 month of simulated sunlight (p = 0.01) and wash 1 showed a significant decrease of 0.039 mg/cm^2^ (54.3%), p = 0.002 (Fig 7).

## Discussion

Insecticide-treated fabrics can reduce vector populations [10,20–22], and reduce the transmission of arthropod borne pathogens [23–28]. All three clothing types tested in this study were effective at providing a high level of personal protection against *Ae*. *aegypti* mosquitoes, demonstrated by the large reduction in biting coupled with the high knockdown and mortality observed up to 5–10 washes. The comparison between the three clothing types revealed similar efficacy between hand dipped and factory-dipped clothing. Despite this similarity, coverage can be inconsistent with hand dipping because it is normally performed by individual users [29] with regular re-application required[20]. It was also noted during this study that the home dipping process caused an odour and change in texture of the clothing. These factors should be considered if home dipping was to be used as a long-term intervention strategy as it could have an influence on consistency of results and on user compliance. The similarity in results between the factory dipped clothing and factory dipped school uniforms is promising for the use of treated school uniforms. The duration of protection provided by the school uniforms was not directly assessed in this study due to availability of the treated school uniforms for testing. As the material and treatment technique were identical we believe the efficacy and duration of protection provided by the school uniforms would be very similar to that provided by the factory dipped clothing however, the school uniform should be tested before being taken forward.

The microencapsulation-treated clothing showed a lower efficacy when compared to the factory and home dipped clothing. The results may be indicative of the different binding methods utilized. Microencapsulation aims to penetrate into the individual fibres of the clothing, unlike polymer coating and hand dipping, allowing a steady and longer lasting release of the insecticide over time [15,30]. This technique may leave less permethrin available on the surface of the clothing and may explain the lower repellency, knockdown and mortality observed. However, this lower level of efficacy may be maintained for longer than the factory dipped clothing which, in the longer term, could offer a more effective clothing type. Unfortunately, after initial testing the manufacturing of this clothing was stopped. Trials are underway to source microencapsulated clothing so it can be fully evaluated as further investigation into the duration of protection provided by microencapsulated clothing may better illustrate the effectiveness of this treatment technique.

The longevity of insecticide-treated clothing varied considerably depending on the wash technique used. The WHO washing appeared to be more rigorous than the machine washing method with the residual permethrin efficacy for the machine-washed clothing being retained for almost double the number of washes. As the mechanical process for machine washing is likely to be far harsher and uses a larger volume of water, the differences identified here could be due to the detergent used for each wash technique. In this study, the detergent used for machine washing was selected because it has no scent and it has been shown not to affect mosquito behaviour and for hand washing, the soap used is recommended by the WHO for evaluating long lasting insecticidal nets [18]. With such clear differences in efficacy between wash techniques, washing technique and detergent used should be considered when designing an intervention using impregnated fabrics. Therefore we recommend that washing of fabrics to determine the duration of protection provided by the clothing, should be performed according to methods that are relevant and representative of the field. HPLC results highlighted a difference in permethrin content between the unwashed fabrics, washed by washing machine and hand washing, before washing (wash 0). This could be due to a variation between batches of clothing but could also be due to variation across a single garment as the samples were taken from the same garment. Previous studies have shown insecticide content within fabrics can vary considerably [31–34]. Although multiple samples were taken for each replicate, this does not account for the variation between different fabrics. Despite this disparity, the effect of the different washing techniques on the permethrin content in subsequent washes is clearly demonstrated.

If clothing is used on a day-to-day basis, the efficacy is likely to drop to sub-optimal levels within weeks of use, primarily due to the effect of washing. We also demonstrate that other factors including ironing will have a significant negative effect. The decrease in efficacy from washing and simulated use has been previously reported with insecticide-treated materials [35–40], where permethrin was also shown to be unstable when exposed to UV light [41] although no significant reduction was observed in this study. The loss of permethrin concentration observed in our studies after washing and ironing exposure could be due to the polymer coating technique leaving greater amounts of permethrin on the surface of the fabric during the treatment process, but a further investigation would be needed for this to be confirmed.

One of the key factors, which was not evaluated here, is the personal protection provided by permethrin treated clothing. This is particularly important when wearing partial coverage clothing (i.e. shorts and short sleeved shirt), which is likely in countries in the tropics and in areas where pyrethroid resistance in mosquito populations is common. These factors are likely to significantly affect the impact of insecticide-treated clothing when worn in a field setting and are being investigated currently by the authors.

For insecticide-treated clothing to have a significant impact on reducing dengue transmission, further work is required to obtain clothing that can withstand washing and environmental exposure for a longer period of time. Solutions to this problem could include replacing clothing after 10–20 washes (5–10 weeks) or using home-dipping permethrin kits, such as the one used in this study and re-treating clothing after the efficacy has dropped to the minimum acceptable level. However, although re-application is easy to perform on a small scale, performing this on a larger community level on a regular basis would be challenging. A cost-effectiveness analysis for an intervention in Thailand suggested a cost greater than $10.6 per child per year would not be of interest to policy makers in Thailand. Using a home dip impregnated kit only once would cost between $5.3-$8 per child per year [42]. Since home-dipping is likely to require more frequent reapplication, this will increase costs beyond the acceptable limit. Nevertheless, if the user compliance issues can be overcome, the home-dipping method may be appropriate for proof of principle studies.

### Conclusion

The clear reduction in the number of bites an individual receives, combined with the high mortality and knockdown caused by permethrin-treated clothing, is proof that insecticide-treated clothing could be a promising additional intervention for dengue prevention. It has the potential to reduce the number of *Aedes* mosquito bites thereby reducing disease transmission. However, for the clothing to be used successfully, improved methods of treatment are needed to ensure duration of protection provided is increased and cost-effectiveness is achieved. A study evaluating field-like conditions would be beneficial to better understand the effect of washing and environmental exposure under natural conditions. In addition, the protection provided by permethrin treated clothing when wearing partial coverage clothing (i.e. shorts and short sleeved shirt) and resistant mosquito populations should be performed.
